# A new basal sauropodiform dinosaur from the Lower Jurassic of Yunnan Province, China

**DOI:** 10.1038/srep41881

**Published:** 2017-02-16

**Authors:** Ya-Ming Wang, Hai-Lu You, Tao Wang

**Affiliations:** 1 School of Earth Sciences and Resources, China University of Geosciences (Beijing), Beijing 100083, P. R. China; 2 Key Laboratory of Vertebrate Evolution and Human Origins of Chinese Academy of Sciences, Institute of Vertebrate Paleontology and Paleoanthropology, Chinese Academy of Sciences, 142 Xizhimenwai Street, Beijing, 100044, P. R. China; 3 College of Earth Sciences, University of Chinese Academy of Sciences, Beijing, 100049, China; 4 Bureau of Land and Resources of Lufeng County, Yunnan Province, 651299, P. R. China

## Abstract

The Lufeng Formation in Lufeng Basin of Yunnan Province, southwestern China preserves one of the richest terrestrial Lower Jurassic vertebrate faunas globally, especially for its basal sauropodomorphs, such as *Lufengosaurus* and *Yunnanosaurus*. Here we report a new taxon, *Xingxiulong chengi* gen. et sp. nov. represented by three partial skeletons with overlapping elements. *Xingxiulong* possesses a number of autapomorphies, such as transversely expanded plate-like summit on top of the neural spine of posterior dorsal vertebrae, four sacral vertebrae, robust scapula, and elongated pubic plate approximately 40% of the total length of the pubis. Phylogenetic analysis resolves *Xingxiulong* as a basal member of Sauropodiformes, and together with another two Lufeng basal sauropodiforms *Jingshanosaurus* and *Yunnanosaurus*, they represent the basalmost lineages of this clade, indicating its Asian origin. Although being relatively primitive, *Xingxiulong* displays some derived features normally occurred in advanced sauropodiforms including sauropods, such as a four sacral-sacrum, a robust scapula, and a pubis with elongated pubic plate. The discovery of *Xingxiulong* increases the diversity of basal sauropodomorphs from the Lufeng Formation and indicates a more complicated scenario in the early evolution of sauropodiforms.

Non-sauropodan basal sauropodomorphs are a diverse and widespread group of herbivorous dinosaurs lived throughout much of Pangaea during Late Triassic to Early Jurassic^1^. However, in China they are mainly known from the Lower Jurassic of Yunnan Province in southwestern China. Since the first basal sauropodomorph, *Lufengosaurus huenei*, was reported in 1941^2^, five genera have been reported from the Lower Jurassic Lufeng Formation in Lufeng Basin^3^,^4^,^5^,^6^,^7^,^8^,^9^,^10^ (Table 1). Recent work has placed *Lufengosaurus* as a member of Massospondylidae and *Yunnanosaurus, Jingshanoaurus* and *Chuxiongosaurus* as basal Sauropodiformes^3^,^11^,^12^,^13^,^14^,^15^,^16^, while the validity of “*Gyposaurus*” has been questioned^1^. Table 2 shows the definitions of clade names adopted here.

Here we report on a new basal sauropodomorph taxon from the Shawan Member of the Lower Jurassic Lufeng Formation. The new material, three partial skeletons buried together, was excavated in Sankeshu Village of Lufeng County in 2013 (Fig. 1). Our morphological and comparative studies show that they represent a single new taxon, and phylogenetic analysis recovers it as a basal sauropodiform. Although basal, this new taxon possesses a derived four-sacral sauropod-like sacrum, a robust scapula, and a sauropod-like pubis, implying complexity in the evolution from basal sauropodiforms to true sauropods.

## Results

SYSTEMATIC PALAEONTOLOGY

Dinosauria Owen, 1842

Saurischia Seeley, 1887

Sauropodomorpha Huene, 1932

Massopoda Yates, 2007

Sauropodiformes Sereno, 2007 (*sensu*^14^)

*Xingxiulong chengi* gen. et sp. nov.

### Holotype

LFGT (Bureau of Land and Resources of Lufeng County, Lufeng, Yunnan, China) -D0002, partial skull and mandible; postcranial skeleton including atlas-axis complex, three cervical vertebrae (possibly C7–C9), seven dorsal vertebrae (possibly D8–D14), complete sacral vertebral series, 35 caudal vertebrae, fragments of ribs and chevrons, left ilium, left pubic apron and distal end of right pubis, proximal end of left ischium and distal portions of articulated ischia, both femora, both broken tibiae and proximal ends of fibulae, left astragalus and calcaneum, putative distal tarsals III and IV, and complete left pes and almost complete right pes.

### Paratypes

LFGT-D0001, articulated postcranial skeleton including axis, complete cervical (C3–C10), dorsal (D1–D14) and sacral vertebral series, 19 anterior caudal vertebrae (possibly Ca3–Ca21), fragments of cervical and dorsal ribs, nine chevrons, right scapula, right ilium, proximal ends of right pubis and ischium, distal portions of femora, distal end of left tibia, and left astragalus. LFGT-D0003, partial skull with mandible and partial postcranial skeleton including eight cervical vertebrae (possibly C3–C10), 14 dorsal vertebrae (possibly D1–D14), nearly complete sacral vertebral series, fragments of ribs and chevrons, both scapulae, both broken humeri, ulnae and radii, partial carpi and manus, right ilium and fragment of left ilium, left pubis and right pubic apron, broken left femur, right tibia and fibula, left proximal ends of tibia and fibula, broken right astragalus and broken distal tarsals, and partial pes.

### Comment

*Xingxiulong chengi* is estimated to have had a total length of 4–5 m and a hip height of 1–1.5 m based on measurements of preserved skeletons (Fig. 2). Among them, LFGT-D0001 is the smallest, and the holotype (LFGT-D0002) is about the same size as LFGT-D0003 (12–14% larger than LFGT-D0001; see Supplementary Information). The fully fused skull elements and neurocentral sutures as well as the relatively large sizes indicate that the holotype and LFGT-D0003 are probably adults, whereas the visible neurocentral sutures and the relatively small size of LFGT-D0001 indicate that it might be a subadult.

### Type locality and horizon

**Figure 1:**
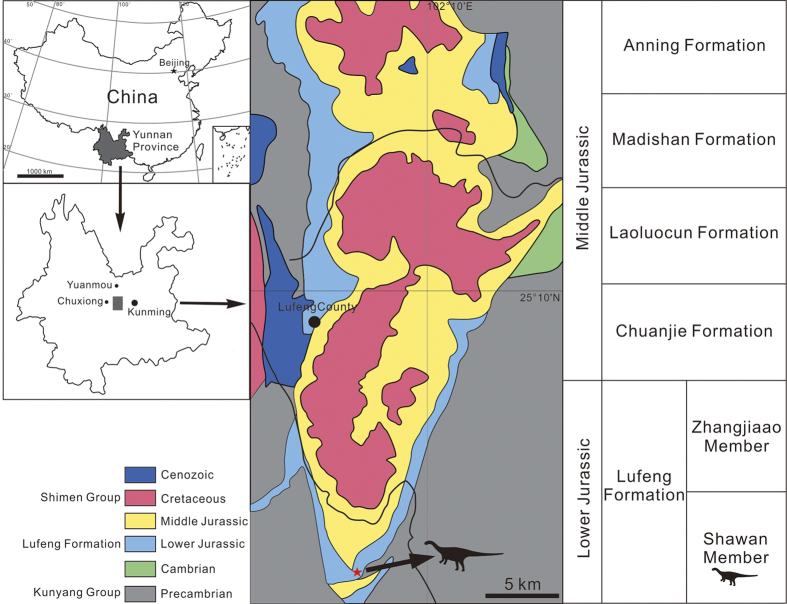
Geographic and geologic map showing the location of *Xingxiulong chengi* gen. et sp. nov. (indicated by the red star and dinosaur silhouette), and generalized stratigraphic section of Early and Middle Jurassic of Lufeng Basin, modified from Fang *et al*.^17^. This map is created by Y.M.W. using CorelDRAW (vers. X7) http://www.corel.com/cn/.

The specimens were excavated near Sankeshu (Three Trees) Village, Jinshan Town, Lufeng County, Chuxiong Yi Autonomous Prefecture, Yunnan Province, southwestern China (Fig. 1). The specimens were from the base of the Shawan Member of the Lower Jurassic Lufeng Formation^17^, composed of dark purple silty mudstones.

### Etymology

The generic name “Xingxiu”(), meaning constellation in Chinese, is derived from the name of the ancient “Xingxiu Bridge” in Lufeng County, which was built during the Ming Dynasty (1368–1644). The specific name is dedicated to Prof. Zheng-Wu Cheng (1931–2015), for his lifetime contribution to Chinese terrestrial biostratigraphy, including the Lufeng Basin.

### Diagnosis

A medium-sized basal sauropodiform with the following unique combination of character states (autapomorphies are marked by *): both surangular and angular extended more anteriorly with respect to the external mandibular fenestra; transversely expanded plate-like summit on top of posterior dorsal vertebrae* (convergent in basal saurischians); four sacral vertebrae, with two primordial sacrals bounded by a dorsosacral and a caudosacral* (convergent in derived sauropodiforms); robust scapula with both ends extremely expanded; ilium with ventral margin of postacetabular process strongly concave*; pubis with elongated proximal pubic plate relative to the pubic apron, with pubic plate approximately 40% of the total length of the pubis*(convergent in basal sauropods); posterolateral process of distal tibia much narrower anteroposteriorly and extended more laterally and distally than anterolateral process*; a median bulge present on the dorsoposterior margin of the astragalus; metatarsal V with strongly expanded proximal end with a proportion of proximal width/total length 0.85*.

### Description

The holotype preserves the posterior part of the skull and mandible as well as the articulated proatlas, atlas, and the anterior end of the axis (Fig. 3a–f). The skull is distorted severely and most of the preserved bones are misaligned. Another skull and mandible is adhered to the anterior caudal vertebrae (C9–C13) of the holotype (Fig. 3g,h). Based on its similar size with that of the holotype and the presence of articulated axis (also present in the smaller specimen LFGT-D0001), this skull and mandible are considered to belong to LFGT-D0003.

In LFGT-D0003, only the posterior three quarters portion of the maxilla, which is the posteroventral part of the ascending ramus and the horizontally directed posterior ramus of the left maxilla, has been remained. The vascular foramina have not been preserved. No ridge is present on the lateral surface of the posterior maxilla, differing from *Lufengosaurus* in which the presence of a maxillary ridge is one of its autapomorphies^18^. Eleven short alveoli are preserved in the maxilla, but their length would be longer due to the covering from the tooth in the mandible (Fig. 3g,h). The lachrymal has a prominent flange ventral to its dorsal end along the anterior margin of the shaft (Fig. 3g,h); a similar condition is also present in *Lufengosaurus, Adeopapposaurus Massospondylus*, and *Riojasaurus*^13^,^18^,^19^,^20^, but absent in *Yunnanosaurus, Jingshanosaurus*, and more derived sauropodiforms. The postorbital has a relatively long contact with the anterior surface of the dorsal ramus of the jugal, similar to that in *Lufengosaurus*. In contrast, the postorbital has a simple tongue-and-groove contact with jugal in *Yunnanosaurus*^21^. The jugal is a triradiate bone and consists of an anterior ramus, a dorsal ramus, and a posterior ramus. The dorsal ramus projects posterodorsally and diverges from the posterior ramus at an angle of approximately 80°, resembling although slightly smaller than that of *Plateosaurus* and *Thecodontosaurus*, whereas it is larger than that of *Lufengosaurus, Jingshanosaurus, Mussaurus* (50°), *Yunnanosaurus* and *Xixiposaurus* (60°), and *Chuxiongosaurus* (70°)^3^,^9^,^21^,^22^,^23^. The quadratojugal is a slender element that gives rise to an anterior ramus and a dorsal ramus (Fig. 3g,h). In lateral view, the two rami diverge from each other at an angle slightly less than 90°, contrary to the angle of 45° in *Lufengosaurus*, 60° in *Yunnanosaurus*, and 110° in *Jingshanosaurus*. The paired quadrates are well preserved in the holotype. Its distal articular surface is composed of a lateral condyle and a medial condyle, which are separated by a sharp ridge (Fig. 3c,d). The lateral condyle is subtriangular in shape, whereas the medial condyle is smaller, semicircular, and situated more ventrally than the lateral one, similar to that of *Lufengosaurus* and *Yunnanosaurus* but differing from that of *Plateosaurus* in which the lateral condyle is more ventrally offset than the medial one. A postparietal fenestra is developed along the suture between the supraoccipital and the parietal (Fig. 3e,f). The supraoccipital slopes anteriorly. The basipterygoid processes are long, slender, and ventrolaterally projected, resembling that of *Plateosaurus*. They diverge from each other at an angle of approximately 80°. In contrast, the basipterygoid processes of *Lufengosaurus* and *Jingshanosaurus* are short, robust, and nearly parallel with each other.

Both the angular and the surangular extend anteriorly beyond the external mandibular fenestra (Fig. 3g,h), resembles the condition in some basal sauropodomorphs such as *Adeopapposaurus* and *Plateosaurus*^24^, but unlike the short extension of these elements in *Lufengosaurus, Yunnanosaurus*, and *Jingshanosaurus*. The articular possesses a large pyramidal process as the medial extension of the glenoid region. Posteriorly, a dorsomedially projected and tab-like process is developed on the medial surface of the retroarticular process (Fig. 3a–d). This feature is also reported to be present in *Coloradisaurus* and is considered as an uncertain autapomorphy^13^. A similar medial process is present in some other basal sauropodmorphs from Lufeng, such as *Jingshanosaurus* and an unnamed new specimen (pers. observ.).

**Figure 2:**
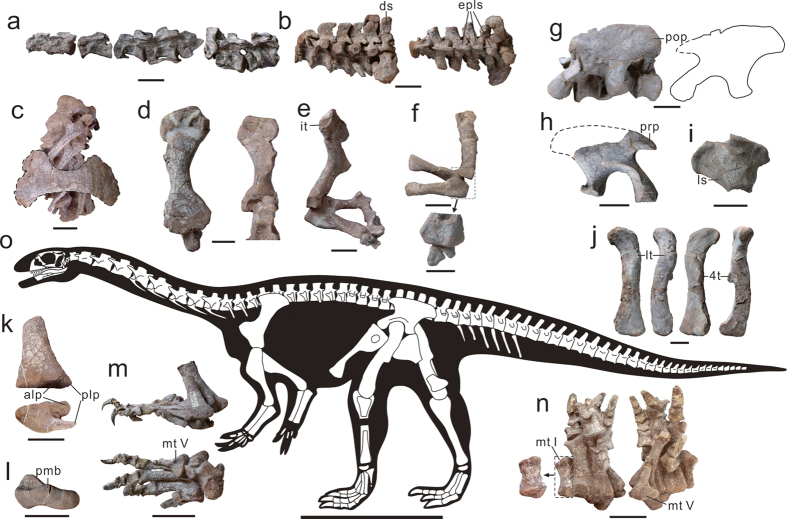
Representative elements of *Xingxiulong chengi* gen. et sp. nov. and reconstruction of the skeleton. (**a**) Cervical vertebrae of LFGT-D0001 (3–10); (**b**) articulated posterior dorsal vertebrae (10–14) and dorsosacral of LFGT-D0001 in lateral and dorsal views; (**c**) scapula with articulated dorsal vertebrae of LFGT-D0003 in left lateral view; (**d**) left humerus of LFGT-D0003 in posterior and anterior views; (**e**) left forelimb of LFGT-D0003 in medial view; (**f**) right articulated humerus, ulna and radius in medial view, and detail of the proximal end of ulna and radius; (**g**) left ilium of LFGT-D0002 (photograph and line drawing) in lateral view; (**h**) right ilium of LFGT-D0003 in lateral view; (**i**) right ischium of LFGT-D0002 in lateral view; (**j**) left femur of LFGT-D0002 in anterior, lateral, posterior and medial views; (**k**) distal end of left tibia of LFGT-D0003 in anterior and distal views; (**l**) left astragalus of LFGT-D0002 in posterior view; (**m**) left pes of LFGT-D0002 in lateral and ventral views; (**n**) right pes of LFGT-D0002 in dorsal and ventral views, with detailed metatarsal I in dorsal view; (**o**) reconstruction of the skeleton of *Xingxiulong chengi* gen. et sp. nov. (scaled to the size of the holotype). Abbreviations: 4t, fourth trochanter; alp, anterolateral process; ds, dorsosacral; epls, expanded plate-like summit; it, internal tuberosity; ls, longitudinal sulcus; lt, lesser trochanter; mt I, metatarsal I; mt V, metatarsal V; plp, posterolateral process; pmb, posterior median bulge; pop, postacetabular process; prp, preacetabular process. Dashed lines represent highlighting (**c**,**b**, and **f**) or reconstruction (**g** and **h**). Scale bars equal 10 cm in (**a**–**n**) and 1 m in (**o**).

**Figure 3:**
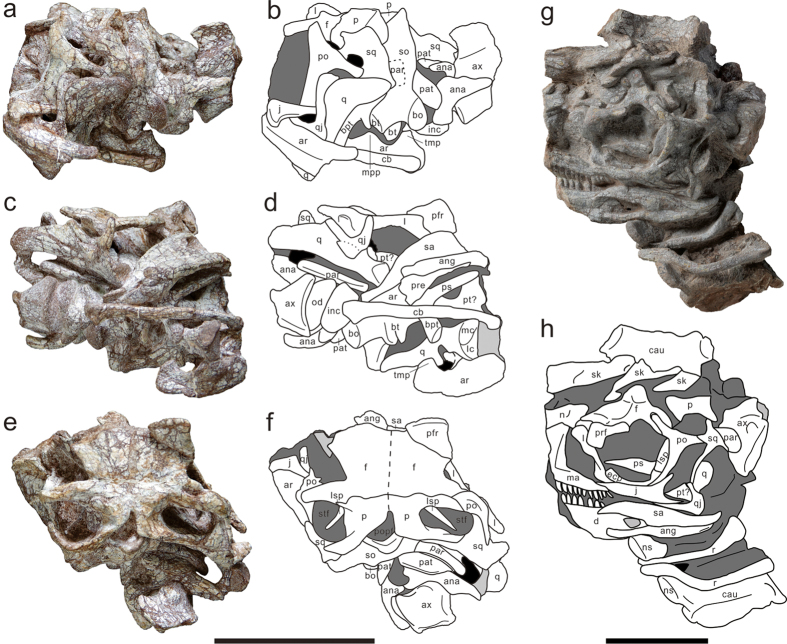
Skull, mandible and atlas-axis complex of *Xingxiulong chengi* gen. et sp. nov. (**a**,**b**) photograph and interpretative drawing of LFGT-D0002 with skull and atlas-axis complex in left lateral and mandible in ventral views; (**c**,**d**) photograph and interpretative drawing of LFGT-D0002 in ventral view; (**e**,**f**) photograph and interpretative drawing of LFGT-D0002 in dorsal view; (**g**,**h**) photograph and interpretative drawing of LFGT-D0003 in left lateral view. Abbreviations: ana, atlantal neural arch; ang, angular; ar, articular; ax, axis; bo, basioccipital; bpt, basipterygoid process; bt, basal tuber; cau, caudal vertebra; cb, ceratobranchial; d, dentary; ecp, ectopterygoid; f, frontal; inc, intercentrum; j, jugal; l, lachrymal; lc, lateral condyle; lsp, laterosphenoid; ma, maxilla; mc, medial condyle; mpp, medial pyramidal process of the articular; n, nasal; ns, neural spine; od, odontoid; p, parietal; par, paroccipital process; pat, proatlas; pre, prearticular; prf, prefrontal; po, postorbital; popf, postparietal fenestra; ps, parasphenoid; pt, pterygoid; q, quadrate; qj, quadratojugal; r, rib; sa, surangular; sk, fragments of skull roof; so, supraoccipital; sq, squamosal; stf, supratemporal fenestra; tmp, tab-like medial process of the retroarticular process. Black fills represent voids within the skull, dark grey fills represent matrix, and light grey fills represent damage. Scale bars equal 10 cm.

Based on overlapping preservation, *Xingxiulong* can be safely said to possess 10 cervical, 14 dorsal, four sacral, and more than 35 caudal vertebrae. Both the proatlases are preserved almost in life position in the holotype (Fig. 3a–f). Anteriorly, they are situated at the dorsolateral corner of the foramen magnum. The atlas consists of the intercentrum, odontoid, and paired neural arches; however, these elements have been distorted and lack critical anatomical details. The centrum of the axis is relatively short with respect to its dorsoventral height, and has moderately compressed lateral and ventral surfaces (Fig. 2a). All cervical centra are proportionally shorter; the length/height ratio of the anterior cervical centra ranges from 2.5 to 3, and this ratio decreases posteriorly, which resembles that of *Lufengosaurus*. This contrasts with *Jingshanosaurus, Massospondylus, Adeopapposaurus*, and *Leyesaurus* with a range of 3–4^25^,^26^,^27^. The ventral keel is present in cervicals 4 to 9 and becomes more prominent along the cervical series, as in *Jingshanosaurus Massospondylus, Adeopapposaurus, Coloradisaurus*, and *Lufengosaurus*. Cervical lamination is poorly developed in *Xingxiulong*; although the prezygodiapophyseal lamina is present in C7 to C9, it is not as developed as in derived sauropodiforms. Neural spine tables are well-developed on the posterior cervicals.

The dorsal vertebrae are slightly amphicoelous, as in other non-eusauropod sauropodomorphs. The blade-like ventral keels are well developed in D1 to D3 and are absent in the middle and posterior series, as seen in most basal sauropodomorphs. The neural spines of the anterior and the last three dorsals are dorsoventrally low, anteroposteriorly short, and expanded to plate-like summits (Fig. 2b). The anterior dorsal neural spine expansion is commonly developed in sauropodomorph dinosaurs; however, it rarely occurs at the posterior dorsal vertebrae, and only developed in some basal saurischians (e.g. *Herrerasaurus* and *Eoraptor*). Hence, we suggest this feature as a possible autapomorphy of *Xingxiulong.* A projecting posterodorsal corner of the neural spine is present in the middle-posterior dorsals, forming a concave posterior margin shared with that of some other basal sauropomorphs but contrasting with the straight posterior margin of *Lufengosaurus, Riojasaurus, Jingshanosaurus*, and *Yunnanosaurus*. Lamination of the dorsal vertebrae is well developed. Notably, a prezygodiapophyseal lamina is developed in D3 to D7 but absent in the posterior dorsals, and the condition in the anterior dorsals is unclear because of the poor preservation. The prezygodiapophyseal lamina is absent in the mid-dorsals in most basal sauropodmorphs, but is present in *Plateosaurus, Sarahsaurus* and *Mussaurus*, and in eusauropods^22^.

**Figure 4:**
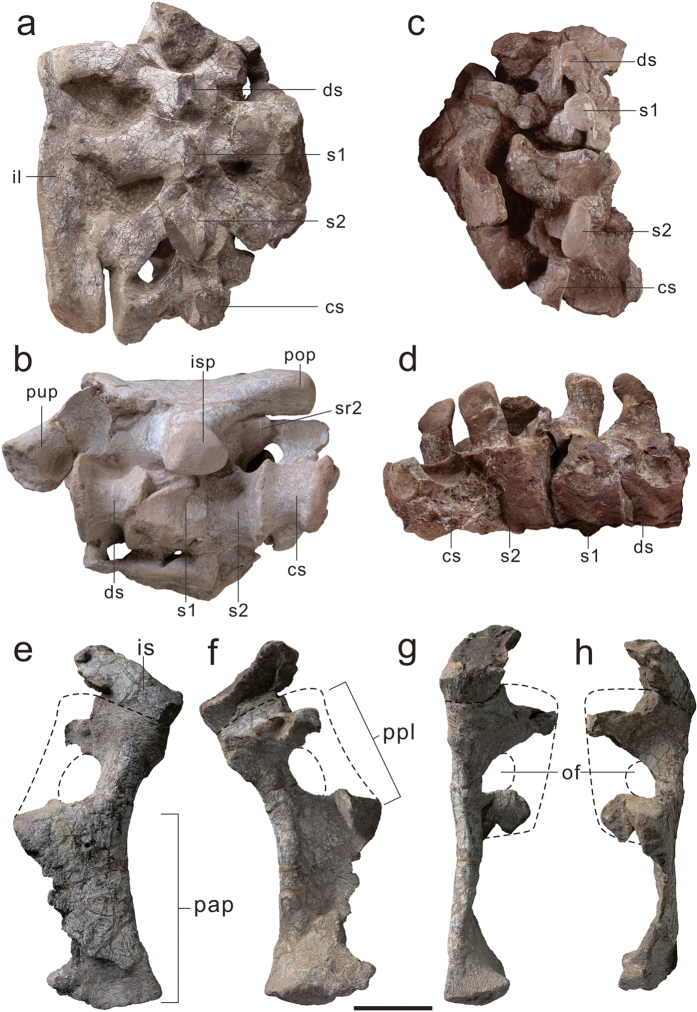
Sacral vertebrae and pubis of *Xingxiulong chengi* gen. et sp. nov. The complete sacral series (LFGT-D0002) in dorsal (**a**) and ventral (**b**) views; the complete sacral series (LFGT-D0003) in dorsal (**c**) and right lateral (**d**) views; left pubis (LFGT-D0003) in anterior (**e**), posterior (**f**), lateral (**g**), and medial (**h**) views. Abbreviations: cs, caudosacral; ds, dorsosacral; il, ilium; is, ischium; isp, ischial peduncle of ilium; of, obturator foramen; pap, pubic apron; pop, postacetabular process; ppl, pubic plate; pup, pubic peduncle of ilium; s1, the first primordial sacral; s2, the second primordial sacral; sr, sacral rib. Scale bar equals 10 cm.

The sacrum consists of four sacral vertebrae (Fig. 4a–d), all of which are preserved in articulation with the ilium. This four-sacral condition is rare in basal sauropodomorphs but resembles that of *Melanorosaurus*^28^, *Leonerasaurus*^29^, and the basal sauropods such as *Barapasaurus*^30^ and *Shunosaurus*^31^. Based on their morphology and relative position, the anterior-most element is interpreted as a dorsosacral, the middle two elements as primordial sacrals, and the posterior one as a caudosacral. The first sacral vertebra (dorsosacral) is placed between the anterior end of the pubic peduncle and the acetabulum of the ilium. It is not fully fused to the subsequent sacral vertebra (first primordial), with the demarcation between them being clear. The transverse process is not entirely fused to the sacral rib; nonetheless, they are sutured tightly with each other to form a single complex that extends anterolaterally and contacts the ilium. The second sacral vertebra (first primordial sacral) is located slightly anterior to the level of the ischial peduncle of the ilium. The third sacral vertebra (second primordial sacral) is placed between the ischial peduncle and the postacetabular process of the ilium. It should be noted, however, that the length of second primordial sacral centrum is approximately equal to the first primordial sacral and the two elements are fused with each other. The transverse process projects posterolaterally, resembles the morphology of most basal sauropodomorphs. The fourth sacral vertebra (caudosacral) is located at the level of the postacetabular process of the ilium. The caudosacral rib is fully fused to the transverse process and extended posterolaterally as the second primordial sacral, forming a lateral expansion to contact the ilium, as observed in *Plateosaurus*. In contrast, the caudosacral rib of *Leonerasaurus* is directed anterolaterally^29^. The neural spines of the sacral series exhibit the same dorsal transversely expansion as in the posterior dorsals.

Based on the preserved elements, *Xingxiulong* possesses more than 35 caudal vertebrae. The caudal vertebrae are tall and robust, and the lateral surface of their centra is concave, as the typical morphology seen in other basal sauropodomorphs (see Supplementary Information). It seems that all the caudal centra have amphicoelous articular facets. The anterior transverse processes are dorsolaterally directed, anteroposteriorly elongated, and dorsoventrally flat, whereas the posterior elements are more slender and nearly horizontal oriented. Lamination of the caudal vertebrae is poorly developed, as in other basal sauropodomorphs; only the prezygodiapophyseal laminae are present in caudal 3 to caudal 8 in LFGT-D0001. The neural spines are tall, posteriodorsally directed, and laterally compressed.

The scapula is characterized by both remarkably expanded proximal and distal ends, with the proximal broader than the distal, as well as a robust scapular shaft (Fig. 2c). The width of the proximal expansion is approximately 56% the total length of the scapula and the distal end is less expanded with a ratio of 49%. A similar robustness is also observed in *Antetonitrus* and *Lessemsaurus*; however, contrary to *Xingxiulong*, the distal end of *Antetonitrus* and *Lessemsaurus* is more expanded than the proximal. A number of non-eusauropod sauropodomorphs (e.g. *Lufengosaurus, Jingshanosaurus, Plateosaurus*, and *Anchisaurus*) has the similar condition to *Xingxiulong* that the proximal expansion is more developed than the distal, although the scapula of them is more gracile^14^,^32^. In contrast, *Yunnanosaurus* displays a scapula with more pronounced expansion of the distal end compared with the proximal end. The scapular shaft is broad, with its minimum width being 19–20% the total length; this proportion is identical to that of *Jingshanosaurus*, whereas greater than that of most sauropodomorphs including some basal sauropods such as *Isanosaurus* and *Shunosaurus*, which have narrower scapular shafts with ratios varying between 15–17%^14^,^32^. *Antetonitrus* and *Lessemsaurus*, however, exhibit a more broadened scapular shaft.

The humerus has a poorly developed internal tuberosity on the medial surface of the proximal end (Fig. 2d,e), differing from the well-developed internal tuberosity in majority of basal sauropodomorphs (e. g. *Adeopapposaurus, Coloradisaurus, Lufengosaurus*, and *Yunnanosaurus*). The length of the ulna is about 61% the length of the humerus (Fig. 2e,f), similar to that of *Yunnanosaurus* and *Jingshanosaurus* but contrasting with *Lufengosaurus* in which this ratio is approximately 68%. Proximally, the ulna is expanded both lateromedially and craniocaudally, with the development of the anteromedial and anterolateral processes. The two processes delimit a shallow radial fossa, resembling that of most basal sauropodomorphs but contrasting with the deep radial fossa in derived sauropodiforms and sauropods. Compared with the ulna, the radius is a slender element (Fig. 2e,f). It is approximately 54% the length of the humerus.

The ilium is similar in overall morphology to that of other basal sauropodomorphs (Fig. 2g,h). The preacetabular process does not project beyond the anterior end of the pubic peduncle. The postacetabular process extends posterolaterally with a subsquare-shaped distal end, contrasting with the pointed postacetabular process of a number of sauropodomorphs. In lateral view, the postacetabular process exhibits a strongly concave ventral margin (between the postacetabular process and the posterior margin of the ischial peduncle), distinguishing it from that of other basal sauropodomorphs. Some taxa such as *Plateosaurus, Massospondylus, Lufengosaurus, Antetonitrus* and *Lessemsaurus* possess an almost straight to slightly convex ventral margin, whereas in other taxa such as *Adeopapposaurus, Jingshanosaurus*, and *Yunnanosaurus* this margin is more convex. Therefore, this feature is a possible autapomorphy of *Xingxiulong*. A posterior projecting heel is present on the distal end of the ischial peduncle of the ilium. The pubis is characterized by a relatively long pubic plate and a short pubic apron, with the former being approximately 40% of the total pubic length (Fig. 4e–h), which has not been reported in other basal sauropodomorph in which generally the pubic plate is short, but resembles the condition in some basal sauropods. In anterior view, the lateral margin of the pubic apron is concave. The distal end of the pubis is expanded both lateromedially and anteroposteriorly, with its anteroposterior depth approximately 16% of the total length of the pubis. The obturator plate of the ischium possesses a longitudinal sulcus on its lateral surface (Fig. 2i).

The femur has a lesser trochanter located distal to the distal margin of the femoral head (Fig. 2j), differing from *Yunnanosaurus* and *Jingshanosaurus* in which the proximal tip is level with the femoral head. In anterior view, the lesser trochanter is placed more close to the mid-line of the mediolateral axis of the femoral shaft with respect to the lateral margin, as seen in most basal sauropodomorphs but unlike the condition in more derived sauropodiforms including *Antetonitrus* and *Melanorosaurus*. The fourth trochanter is located close to the centre of the posterior surface of the femur, contrasting with the more medially placed fourth trochanter in *Lufengosaurus, Adeopapposaurus, Coloradisaurus, Riojasaurus* and more derived taxa (e.g. *Anchisaurus, Aardonyx, Lessemsaurus and Antetonitrus*)^27^. The distal end of the tibia possesses a posterolateral process that is much narrower anteroposteriorly and extends more laterally and distally than the anterolateral process (Fig. 2k); in contrast, the posterolateral process of other basal sauropodomorphs is similar in width with or slightly narrower than the anterolateral element and they almost share the same lateral extension (e.g. *Plateosaurus, Massospondylus, Coloradisaurus, Lufengosaurus*, and *Jingshanosaurus*) or the former projects less laterally than the latter (e.g. *Sarahsaurus, Yunnanosaurus, Mussaurus*, and sauropods). The posterior surface of the astragalus bears a dorsally convex bulge close to the mid-line that is lower than the ascending process (Fig. 2l). This feature is also reported in *Mussaurus*, which has been suggested to be one of the autapomorphic features of this taxon^22^; the bulge of *Xingxiulong* seems to be less developed and located more laterally than in *Mussaurus*, although it is still more pronounced than that of *Plateosaurus* and *Blikanasaurus*. All the pedal elements are well preserved in the holotype (Fig. 2m,n). Metatarsal I is a robust element; its mid-shaft is mediolaterally wider than that of other metatarsi (see Supplementary Information). Metatarsal V has a markedly expanded proximal end, with its width being approximately 85% of the total length and forming a triangle profile. This differs from other sauropodomorphs including derived taxa in which this ratio ranges from 50% to 77%^15^. Although it appears that metatarsal V of *Antetonitrus* has the same proximal expansion; unfortunately, the lack of its distal end precludes the comparison between the two taxa. Hence, we assume this feature as a potential autapomorphy of *Xingxiulong*. The pedal phalangeal formula is 2-3-4-5-1, as in other basal sauropodomorphs.

## Discussion

**Figure 5:**
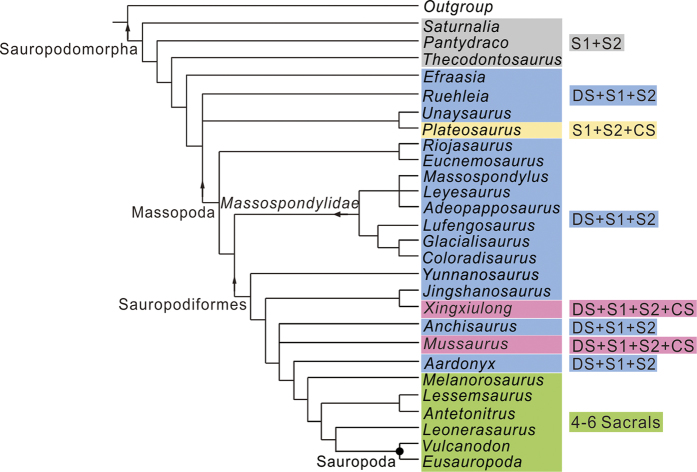
Abbreviated reduced consensus tree of the phylogenetic analysis illustrating the phylogenetic position of *Xingxiulong chengi* gen. et sp. nov. and evolutionary history of basal sauropodomorph sacral vertebrae (right). Abbreviations: DS: dorsosacral; S1 and S2: two primordial sacrals; CS: caudosacral.

The strict consensus tree obtained from the phylogenetic analysis shows a large polytomy formed by most of basal sauropodiforms (see Supplementary Information). Alternatively, the reduced consensus tree in which the fragmentary *Blikanasaurus* was pruned from the MPTs displays a high degree of resolution and resolves *Xingxiulong* as a basal sauropodiform (Fig. 5). Moreover, *Xingxiulong* has a sister-taxon relationship with *Jingshanosaurus*, which is supported by five unambiguous synapomorphies: anterior margin of the infratemporal fenestra placed behind the orbit (character 57.0); width of the scapula greater than 20% of its length (character 202.0); lateral margin of the pubic apron concave (character 267.1); anteroposterior expansion of the distal pubis greater than 15% of the total length (character 270.2); angle between the long axis of the femoral head and the transverse axis of the distal femur about 30 degrees (character 285.0). Alternative positions of *Xingxiulong* were tested to evaluate its suboptimal position. Placing *Xingxiulong* to a more basal location as a member of Massospondylidae implies 5 extra steps, placing *Xingxiulong* more basal to *Yunnanosaurus* but more derived than Massospondylidae implies 6 extra steps, and forcing it to a position more derived than *Anchisaurus* but basal to *Aardonyx* requires 8 extra steps. Trees depicting this new taxon as a position more derived than *Jingshanosaurus* but basal to *Anchisaurus*, or between *Yunnanosaurus* and *Jingshanosaurus*, both require 3 extra steps. However, placing *Xingxiulong* as a basal member of Massapoda that is more basal to (Massospondylidae + Sauropodiformes) only requires 2 steps. This is unsurprisingly given some primitive features present in *Xingxiulong* when compared with Massospondylidae and Sauropodiformes, such as the dorsal margin of postorbital gently curved (character 54.0), anterior margin of the infratemporal fenestra behind the orbit (character 57.0), and presence of a medial process of the articular behind the glenoid (character 104.0). This much more basally suboptimal position of *Xingxiulong* suggests a high level mosaic evolution around the beginning of basal Massopoda and basal Sauropodiformes. Nonetheless, the most parsimonious position of *Xingxiulong*, which is a basal sauropodiform, is adopted here, although more material and further studies are needed in future to test its alternative phylogenetic placement.

Before the discovery of *Xingxiulong chengi*, members of three Sauropodomorpha groups have been recognized in the Lower Jurassic of Lufeng Basin: one massospondylid (*Lufengosaurus*), three basal sauropodiforms (*Yunnanosaurus, Jingshanosaurus*, and *Chuxiongosaurus*), and a putative sauropod^33^. *Chuxiongosaurus* was excluded from this phylogenetic analysis due to its controversial status as it is possibly synonymous with *Jingshanosaurus* (pers. observ.). The discovery of *Xingxiulong* adds another basal sauropodiform, and demonstrates the close relationships among these Lufeng basal sauropodiforms. This shows that there were two evolutionary clades among Lufeng basal sauropodomorphs: one as a member of Massospondylidae, while all others on the Sauropodiformes clade, especially at its base, indicating the Asian origin for this clade.

**Figure 6:**
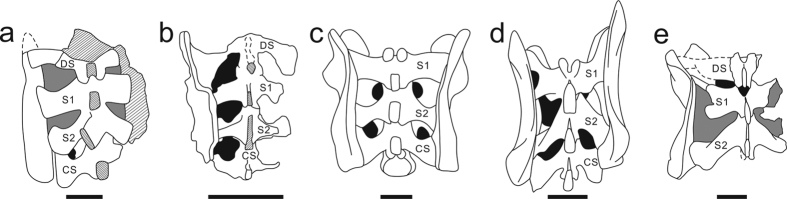
Comparison of sacral vertebrae of *Xingxiulong chengi* gen. et sp. nov. and other basal sauropodomorphs (in dorsal view). (**a**) *Xingxiulong chengi* (four sacrals; LFGT-D0002). (**b**) *Leonerasaurus taquetrensis*, a relatively derived non-sauropodan sauropodiform (four sacrals; MPEF-PV 1663, redrawn from Pol *et al*.^29^). (**c**) *Plateosaurus engelhardti*, a non-massopod sauropodomorph (three sacrals; the holotype UEN 552, redrawn from Galton^43^). (**d**) *Plateosaurus trossingensis*, a non-massopod sauropodomorph (three sacrals; the holotype SMNS 13200, redrawn from Galton^43^). (**e**) *Efraasia minor*, a non-massopod sauropodomorph (three sacrals; SMNS 17028, redrawn from Yates^44^). Dashed lines represent reconstruction, slash lines represent damage, black fills represent voids within the sacrum, and grey fills represent matrix. Abbreviations: DS: dorsosacral; S1 and S2: two primordial sacrals; CS: caudosacral. Scale bars equal 10 cm.

Interestingly, although relatively basal, *Xingxiulong* possesses a sacrum composed of four sacral vertebrae, with two primordial sacrals bounded by a dorsosacral and a caudosacral. This condition is contrary to the three-sacral sacrum in most basal sauropodomorphs including other Lufeng taxa (Fig. 6). A key step in the transition from basal sauropodomorphs to sauropods is the increase of sacral vertebrae, and a four-sacral sacrum is traditionally considered as one of the diagnosis of sauropods^29^,^34^. Three major stages in the evolutionary history of the sauropodomorph sacrum were summarized by Pol *et al*.^29^: first, a sacrum composed of two primordial sacrals in the early stage; second, a sacrum composed of three sacrals with the incorporation of a dorsosacral in most basal sauropodomorphs (except for *Plateosaurus* in which the third element is a caudosacral); third, a sacrum composed of four sacrals characterized by the incorporation of a caudosacral present in Sauropoda. Intriguingly, a new basal sauropodiform (*Leonerasaurus*) reported by Pol *et al*.^29^ also displays a four-sacral sacrum and this taxon is closely related to Sauropoda in our analysis (also McPhee *et al*.^15^); therefore, the four-sacral condition is also diagnostic of close relatives of Sauropoda. Consequently, *Xingxiulong* is the most basal sauropodomorph with a four-sacral sacrum, which may be also convergently achieved later in *Mussaurus*^22^, and then achieved in Sauropoda and its close relatives.

Moreover, *Xingxiulong* has a relatively long pubic plate that is approximately 40% of the total pubic length. Generally, the pubic plate is short, being 25% of the total length of the pubis in other basal sauropodomorphs (e.g. *Plateosaurus, Massospondylus, Adeopapposaurus, Coloradisaurus, Lufengosaurus* and *Yunnanosaurus*)^27^. In contrast, in sauropods the pubic plate is approximately 33% of the pubic length, and this ratio increases to 45–50% in camarasauromorph sauropods^35^,^36^,^37^. However, the distal pubic apron twisted posteromedially in sauropods, unlike the transversely oriented and compressed pubic apron in *Xingxiulong* and other basal sauropodomorphs^37^. Nonetheless, the relatively long pubic plate in *Xingxiulong* distinguishes this taxon from other basal sauropodomorphs and resembles that of sauropods.

It also should be noted that the femur of *Xingxiulong* is robust because the mediolateral width of its femoral shaft (9.4–10.1 cm; see Supplementary Information) is greater than that of most other basal sauropodomorphs although its femur is proportionally shorter^29^,^38^. Meanwhile, compared with other basal sauropodomorphs, metatarsal I of *Xingxiulong* is remarkably robust. The minimum width of the mid-shaft of metatarsal I is 38% of its length (left element; 44% in right element), and it is the transversely widest element among all the metatarsi, which resembles that of *Aardonyx, Antetonitrus* and *Blikanasaurus* and is a sauropod-like feature^39^,^40^. In addition, *Xingxiulong* has a robust scapula and metatarsal V in comparison with other basal sauropodomorphs. All these morphological features as well as the four-sacral condition indicate that *Xingxiulong* probably had a large body mass and an increasing gut volume, although it only achieved a medium body size relative to other non-sauropodan sauropodomorphs (e.g. *Plateosaurus, Riojasaurus*, and *Jingshanosaurus*). However, the relationship between the large gut-capacity and the gigantism of the body size remains to be studied further.

These intriguingly features that *Xingxiulong* displays inevitably raise questions about its preferred locomotory habit. As noted by Yates *et al*.^39^, the habitual quadrupedalism is supported by modifications such as the increase of relative length of the forearm relative to the hindlimb, the presence of a large anterolateral process on the ulna, and the straightening of the femoral shaft. None of these specializations is present in *Xingxiulong*; its ulna and femur exhibit the general features as occurred in other basal sauropodomorphs instead of the quadrupedal clade. Recently, McPhee *et al*.^41^ suggested that the massive scapula of some non-sauropodan sauropodiforms possibly provides two utilities: to counteract the shear stresses produced by the large-bodied quadruped with the less erected forelimb and to increase the mobility of the forelimb for bipedal high-browsing. The three Lufeng basal sauropodiforms, (*Xingxiulong, Jingshanosaurus*, and *Yunnanosaurus*) share the robust scapula as well as a number of features involving gigantism (although it remains questionable for *Yunnanosaurus* as it is probably a sub-adult or juvenile; pers. observ.). They might represent an important stage towards obligate quadrupedal and further biomechanical analysis on the locomotory habit of these taxa is critical to investigate the scenario at the beginning of the sauropodiforms.

*Xingxiulong* provides additional information to understand the evolutionary history of basal sauropodomorphs. Many convergent features shared by *Xingxiulong* and Sauropoda imply that the evolution of the pelvic and pes decoupled from many other features in the transition to the sauropods, such as the shortening and heightening of the skull, the shortening of the trunk, and most importantly, and the increase of body size, which shows a more complex and homoplastic evolution of basal sauropodomorphs than we previously thought. Nonetheless, the unique suite of characters of this new taxon highlights the need for more well-preserved fossils and a more comprehensive analysis to elucidate the interrelationships of basal sauropodomorphs and to improve our knowledge on the origin of Sauropoda.

## Methods

A phylogenetic analysis was performed based on the data matrix published by McPhee *et al*.^41^ in order to determine the phylogenetic affinities of *Xingxiulong* within Sauropodomorpha. We choose this data matrix because it represents one of the most comprehensive datasets for basal sauropodomorphs up to now. A matrix of 365 characters and 55 taxa was analyzed using TNT1.1^42^, applying a heuristic search retaining 10 shortest tree from every 1000 trees, followed by an additional round of tree bisection reconnection (TBR) branch swapping. The following characters were treated as ordered: 8, 13, 19, 23, 40, 57, 69, 92, 102, 117, 121, 131, 144, 147, 149, 150, 157, 162, 167, 170, 177, 205, 207, 225, 230, 237, 245, 254, 257, 270, 283, 304, 310, 318, 338, 351, 354, 356, 361, 365. The phylogenetic analysis produced 648 most parsimonious trees, with tree length of 1274 steps (CI = 0.338, RI = 0.669).

## Additional Information

**How to cite this article**: Wang, Y.-M. *et al*. A new basal sauropodiform dinosaur from the Lower Jurassic of Yunnan Province, China. *Sci. Rep.* **7**, 41881; doi: 10.1038/srep41881 (2017).

**Publisher's note:** Springer Nature remains neutral with regard to jurisdictional claims in published maps and institutional affiliations.

## Supplementary Material

Supplementary Information

## Figures and Tables

**Table 1:** Basal sauropodomorphs from the Lower Jurassic Lufeng Formation of the Lufeng Basin, Yunnan Province, China.

Taxon	Publications
*Lufengosaurus huenei*	Young^2^,^5^; Barrett *et al*.^18^; Sekiya and Dong^9^
*Lufengosaurus magnus*	Young^5^
*Yunnanosaurus huangi*	Young^4^,^7^; Barrett *et al*.^21^
*Yunnanosaurus robustus*	Young^7^
“*Gyposaurus*” *sinensis*	Young^3^,^6^,^7^
*Jingshanosaurus xinwaensis*	Zhang and Yang^8^
*Chuxiongosaurus lufengensis*	Lü *et al*.^12^

**Table 2:** Clade names used in this study.

Clade	Definition	Source
Sauropodomorpha	The most inclusive clade containing *Saltasaurus* but not *Passer* or *Triceratops*	Sereno^11^
Plateosauridae	The most inclusive clade containing *Plateosaurus engelhardti* but not *Saltasaurus*	Yates^45^
Massopoda	The most inclusive clade containing *Saltasaurus* but not *Plateosaurus engelhardti*	Yates^45^,^46^
Massospondylidae	The most inclusive clade containing *Massospondylus* but not *Plateosaurus engelhardti* or *Saltasaurus*	Sereno^11^
Sauropodiformes	The most inclusive clade containing *Saltasaurus* but not *Massospondylus*	McPhee *et al*.^15^
Sauropoda	The least inclusive clade containing *Vulcanodon* and *Saltasaurus*	Salgado *et al*.^35^ Langer *et al*.^47^
Eusauropoda	The least inclusive clade containing *Shunosaurus* and *Saltasaurus*	Upchurch *et al*.^37^
