# Supplementary material for: A new basal sauropodiform dinosaur from the Lower Jurassic of Yunnan Province, China
Source: Sci Rep. 2017 Feb 16;7:41881. doi: 10.1038/srep41881 (PMC5312170; doi:10.1038/srep41881)
Supplement: Supplementary Information [file srep41881-s1.pdf]

SUPPLEMENTARY INFORMATION

A new basal sauropodiform dinosaur from the Lower Jurassic of Yunnan Province, China

Ya-Ming Wang[1], Hai-Lu You[2,3 *], Tao Wang[4]

[1] *School of Earth Sciences and Resources, China University of Geosciences (Beijing), Beijing 100083, P. R. China*

[2] *Key Laboratory of Vertebrate Evolution and Human Origins of Chinese Academy of Sciences, Institute of Vertebrate Paleontology and Paleoanthropology, Chinese Academy of Sciences, 142 Xizhimenwai Dajie, Beijing, 100044, P. R. China.*

[3] *College of Earth Sciences, University of Chinese Academy of Sciences, Beijing, 100049, China.*

[4] *Bureau of Land and Resources of Lufeng County, Yunnan Province, 651207, P. R. China*

[*] Correspondence and requests for materials should be addressed to H. L. Y. (email: youhailu@ivpp.ac.cn)

1) Supplementary figure S1: Skeletons of *Xingxiulong chengi* gen. et sp. nov. *in situ*.

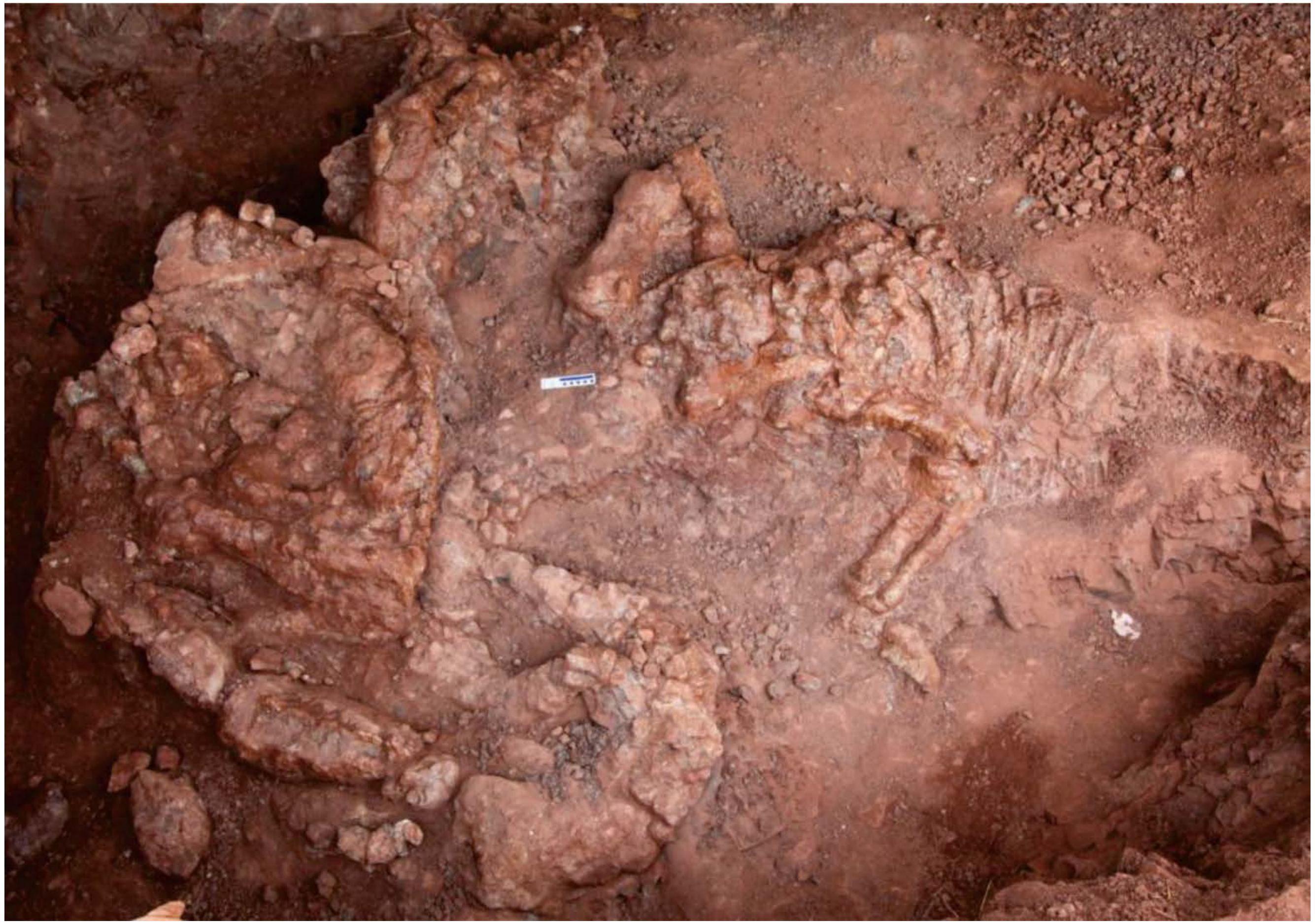

2) Supplementary figure S2: vertebral column of LFGT-D0003. a, complete dorsal vertebrae and a dorsosacral; b, the posterior three sacral vertebrae; c, anterior caudal vertebrae. Scale bar equals 10 cm.

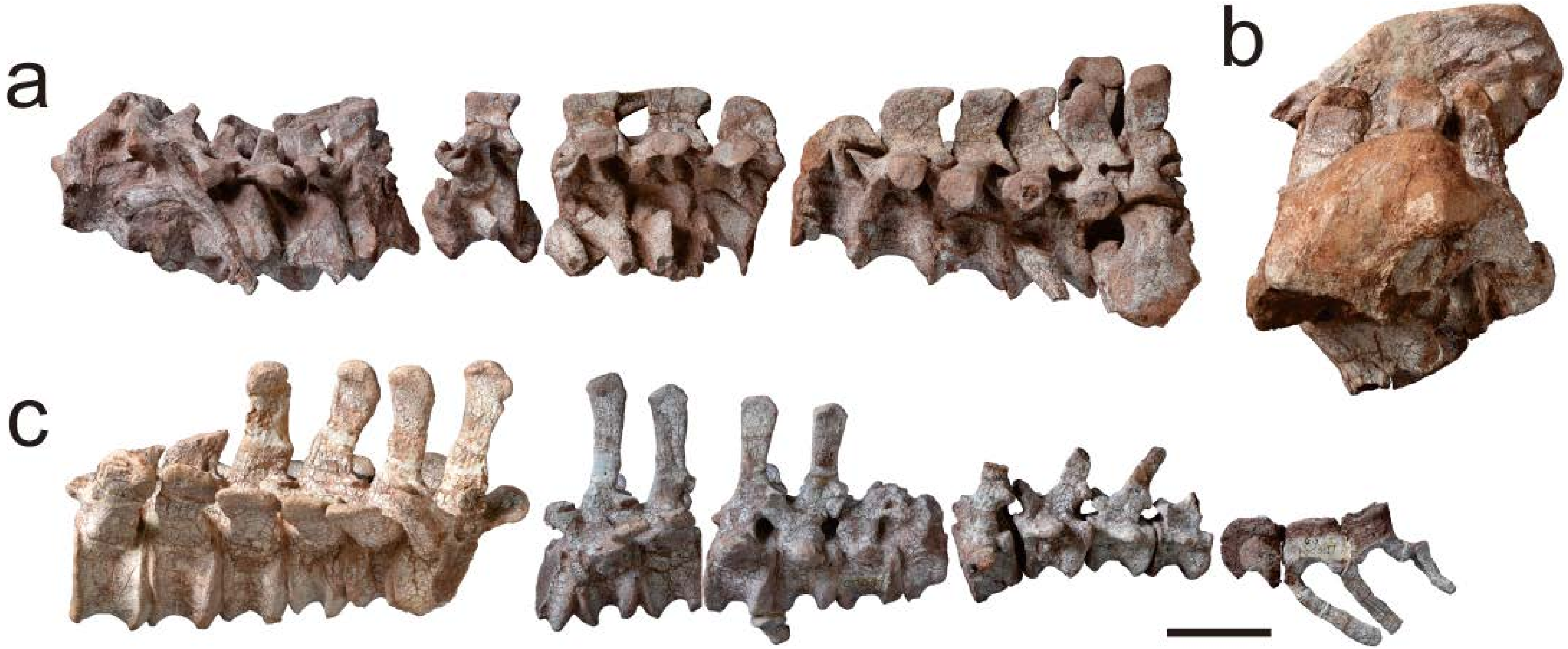

3) Supplementary figure S3: strict consensus tree of phylogenetic analysis. Numbers below the nodes represent bootstrap values higher than 50% (left) and Bremer support values higher than 1 (right).

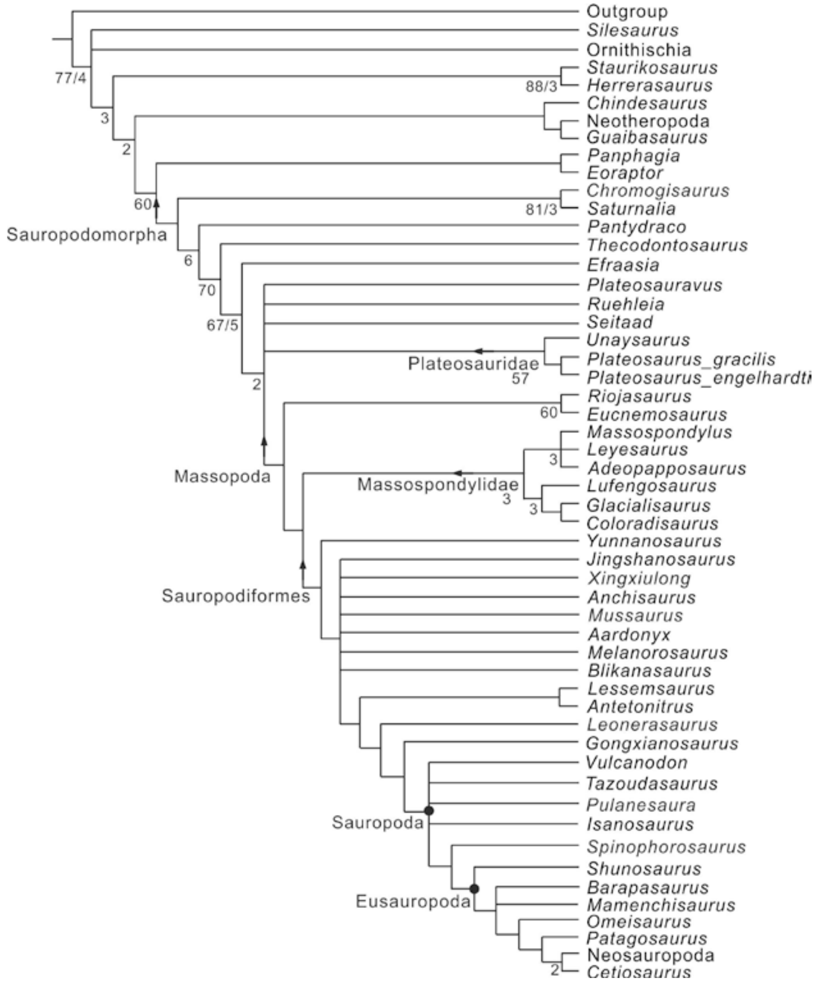

4) Character states for *Xingxiulong chengi* used in the phylogenetic analysis in this study. See McPhee *et al*.[1] for character list and states for remaining taxa included in this analysis.

Xingxiulong chengi

??????????????????????1???1?1111???0?10?0????0110011001100000000101?01?00100101?00?101???????0?????????001??000?????????1100
111100100110001?011010002001110011000000000100001100110???1110000001?????00?0000???1120?011100????1??????1101????????????
??001001311000010?1011002??11??00010000000111110000101101010?00000[01]10111001001101000?01011000110111000001?21001100002
1002

5) Supplementary Table S1: measurements (cm) of *Xingxiulong chengi* gen. et sp. nov. (LFGT-D0001, 002 and 003). Abbreviations: C, centrum; CrF, cranial face of the centrum; CaF, caudal face of the centrum; DI, diapophysis (mediolateral length); NS, neural spine (height: measured from the lower margin of the postzygapophysis to the top of the neural spine; length: measured as the craniocaudal dimension at midspine height); PRZ, prezygapophyses; POZ, postzygapophyses; TP, transverse processes (mediolateral length and anteroposterior width). * represents estimated value.

Cervical vertebrae

Element	Height	C length	C Height	CrF Height	CrF Width	CaF Height	CaF Width	NS Length	NS Height	NS width(top)	PRZ Width	PRZ Length	POZ Width	POZ Length
LFGT-D0001														
Axis	6.5*	8.3*	3	2.6	-	3.5*		3.9						2.2
C3	7.2	10.3	3.7	3.4		4.8*		5	1.3			6		2.9
C4	8.6	11.2 *	4	4.3		5.1	4.9	6	1.7					
C5	9.2	12*	4.2	4.9	4.6	6*	5.6*	5.3	1.8				2.7	4.3
C6	10.5	11.8	4.4	5.5*	5.4	6.6	5.7	5.1	1.9			7	2.9	4.5
C7												8.1		
C8	12.7	11.4	5.5			6.5		3.8	2.8			5.6		4.8
C9	12.9	9.4	5.8	6.2				2.8	3.2	2		5.5		5
LFGT-D0003														
Axis	9.4						3.2		1.5				2	2.9
C3		12.7					3.3	6.3			2.5	6.5	2.3	3.5
C4												6.6		
C5		13.5												

C6	13.3				7.1	6.8								
C7													4.2	4.9
C8												7.7		
C9	13.6	4.2			7.7	8.5						7.3		

Dorsal vertebrae

Element	Height	C length	C Height	CrF Height	CrF Width	CaF Height	CaF Width	DI length	NS Length	NS Height	NS width(top)	PRZ Length	POZ Length
LFGT-D0001													
D1	14.3	7.9	5.7	6.1		6.5		3.2	3	2.9	2.7		3.3
D2	13.7	7.6	6	6.3		6.9		3.3	3.1	2.8	2.6	3.9	3.5
D3	13.1	7.8	6.1	6.7		7.4		3.5	3	3.2	2.5		
D4	13.3	6.2	6	7.1		7.6		3.9	3.2	3.4	2.4		
D5	14.2	6.8	5.4	7.2				4.5	3.5	4.4	2.2		
D6	14.3	6.9	5.7	7	6.4	7.4	6		4.2	4.3	1.2		
D7	13.9	7	5.6	7.2	5.8	8		5.1	5.1	4.5	0.6		
D8	14.3	5.7	5.6	8		8.1		5.6	5.2	4.5	0.5	3.2	
D9	14.5	5.6	5	8.1		8.2	7.4	5.8	5.7	4.5*	0.6*	3.3	
D10		6.2*	5.5			8.4							4
D11	15.8	7	6	9		8.2		5.8	6.1	5	1.1		2.7
D12	16.4	7.1	6.2	8.5		8.4		6	4.8	5.5	1.4		2.5
D13	16.8	7.4	6.4	9.4		9.5		5.8	5.5	5.7	2.4		2.5
D14	17	7.2	7	10		10.1		4.4	5	6.2	2.7		2.4
LFGT-D0002													
D6		8.3	5.8	5.8	5.3*	8	8						
D7		8	5.8	7	8.3	8.1*	8.7	4.5					
D8		7	6.2	7.3	8.1	9.8	8.4*						

D9		7.5	7.5	8.4	8.5	9.9	8.6					
D10		8	6.7	8.8	9.4	9.8	10.1	6.8				
D11	13.2	8.2	6.8	8.3	10.1		10.7	6.1	7.5	6.7		
D12	13.4	8.4	7.2		11.3*	11	12.7	8.2	7.5	6.8	0.8	
D13		8.9	7.1	10.1	12.7							
D14		8	7.6			10.9	13.6					
LFGT-D0003												
D1		10.1							4.5	3.1	3.5	6
D2		8.26							2.7	3.4		
D3		8.7*							2.7	4.2	4.8	
D4		8							3.7	4.4	3.5	
D5		7.9							4.2	4.5	1.9	
D6		8.1				6.6	8.1		5	5.2		
D7		8.4	5.3			8*	8		5.7	4.9	0.8	
D8									7.1	5.9	0.8	
D9		8.9	6.2		9.3		11.7	5.5	7.2	6.1	0.9	
D10		9.4	6.5		10.7		11.6	4.8	7	6.7	1	
D11		9.2	6.2		12.9		13.4	4.6	7.2	7.7	1.1	
D12					12.8*			4.2*				

Sacral vertebrae

Element	Height	C length	C Height	CrF Height	DI length	NS Length	NS Height	NS width(top)
LFGT-D0001								
S1	18	6.8	6.7	10.1	4.1	3.9	6.5	3.5
S2								3.2
LFGT-D0002								
S1		9.6						3.8
S2		7.3*				5.3		
S3		8.1				6.2		4.1
S4		8.2				6		4.1
LFGT-D0003								
S1						5.5		2.5
S2						5.1		3.8
S3						5.4		3
S4						5.1		

Caudal vertebrae

Element	Height	C length	C Height	CrF Height	CrF Width	CaF Height	CaF Width	TP length	TP width	NS Length	NS Height	NS width(top)	PRZ Length	POZ Length
LFGT-D0001														
C3		6.3	7.5	9.2	7.8	8.5		8.6	4.4					2.6
C4		6.2	7.4	9.0		8.4		9.2	4				3.3	2.7
C5	22.2	6.0	6.6	9.0		8.1		9.8	3.9	4.4*	10.8*	1.8	3.2	2.8
C6	21.8	6.2	6.4	8.5		8.0		9.8	3.5	3.1*	11.5*	1.7	3.2	2.7
C7	20*	6.2	5.2	8.3		7.2		9.9	3.5	2.8*	11*	1.4	3.3	2.6
C8	20.4	5.9*	5.1*	7.8		7.5		8.4	3.4*	2.6*	11.1*	1.6*	3.2	
C9	20.2	5.9	5.1	7.7		6.9		7.8	3.3*	2.3	11.4*	1.5	3.0	2.2
C10	18.7	5.8	5.2	7.1		6.8	7.0	7.0	3.0	2.4	10.3*	1.1	2.7	
C11	18.3	6.0	4.8	6.3	7.2	6.7		6.9	2.8	2.4	9.4	1.0		2.0
C12	17.2	5.4	4.6	6.2		6.5		5.9	2.6	2.2	8.2	1.2		
C13		5.2	4.5	6.0		6.0								
C14				5.3										
C15		5.0	4.6	5.3	5.0	5.2	4.9	3.6	2.2					2.3
C16		5.0*	2.8	5.5	5.1	4.8	4.7	3.6	2.4					
C17	10.6	4.8*	2.7	5.1	4.9	4.7	4.6	3.5	2.0	1.8	5.9	0.7		
C18		4.8	2.7	5.0	4.8	4.3	4.2							
C19		5.2*	3.4*						1.5					
C20		4.4	2.9						1.2					
C21		4.2	2.6											
LFGT-D0002														

C1	7.3	11.1		11.2	10.9		
C2	8.2	11.2	12.8	11.6	10.9		
C3	7.7	10.9	11.5	11	10.5		
C4	8.3	10.7	11.2	10.5	10.3		
C5	7.9	10.6*	9.7	10.2	9		
C6	7.5	9.4	8.6	9	8.8		
C7	6.8	9.5	8		7.8		
C8	6.1	8.4	8		8.3		
C9					8.1		
C10	4.6*					3.6	
C11	5.2					3.7	2.4
C12	6.3	7.2*		7		3.7	2.4
C13	6.2	6.2		6.9		3.8	
C14				8.9	7.5		
C15	5.4	8.4	7.5	7.8	6.9		
C16		7.2*	7.2				
C17	5.2	6.2	4.9	5.5*	5.5		
C18	5.1	5.6	5.5	5.3	5.7		
C19	5.9	5.5	5.6	6	5.2		
C20	5.8	5.4	5.2				
C22	4.7	4.6	5.3	5.2	5.1*		
C24				5.5	5.6		
C25				5.8	5.8		

C26	4.8		5	5	4.9
C27	5.3*	5.1	4.8	4.9	4.7*
C28	5.1	4.5	4.3	4.5	4.1
C29	5.3*				
C30	4.8			5	4.2*
C31	5.6*	4.7	4		
C32	5.2		4.1	3.2	3
C33	5				
C34	3.6		3.9		2.9
C35		3.6	3.2		

Chevron

LFGT-D0001

Element	Length	Maximum anteroposterior width of the distal blade	Transverse width of the proximal end
1	13.8	1.7	4.9
2	17.8	2	5.4
3	19.5	2.5	4.9
4			5.1*
5			5.4*
6		2.5*	4.2

Pectoral girdle

Element	Total Length	Width of the anterior end	Least width of scapula blade	Distal height of scapula blade
LFGT-D0001				
Left scapula	41.5*	18.5*	7.8	16.9
LFGT-D0003				
Left scapula	43.5	24.2	8.6	21.3
Right scapula			8.7	

Humerus

LFGT-D0003

Element	Total Length	Deltopectoral crest length	Proximal width (mediolateral)	Humeral shaft minimus width	Distal (condyles) width
Left humerus	39.5	19.3	18.3	6.3	14.7
Right humerus					15.2

Ulna and radius

LFGT-D0003

Element	Total length	Proximal maximum anteroposterior length	Proximal maximum mediolateral width	Mid-shaft minimum transverse width	Mid-shaft anteroposterior length	Distal (condyles) maximum transverse width	Distal anteroposterior length
Left ulna	24.1						
Right ulna	24.3	10.3	6.1	2.8	3.8	3.1	7.7
Left radius	21.5						
Right radius	23.8*	8.4	4	3.4*	3.6*	4.4	6.2

Metacarpals

LFGT-D0003

Element	Total length (medial/lateral)	Proximal width (mediolateral)	Proximal height (dorsoventrally)	Distal width (mediolateral)	Distal height (dorsoventrally)
Left metacarpal I	6.3/7.8	6.2*	3.5	5.5	3.2
Left metacarpal III	7.7	2.2	3.6	3.4	2.4
Right metacarpal II				2.7	2
Right metacarpal III	7.1*				
Right metacarpal IV	5.7	2.7	1.9	1.9	

Ilium

Element	Total length (dorsal margin)	Total height	Anteroposterior length acetabulum	Length of the preacetabular process	Height of the preacetabular process	Length/width of the pubic peduncle	Length/width of the ischium peduncle	Length/height of postacetabular process
LFGT-D0001 right		27.5	16.3			15.2/6.9	9.0/-	14.9*/13.8
LFGT-D0002 left						15/7.8	9.2/8.2	13.8/10.5
LFGT-D0003 right			12.2	6.8	5.9	13.4/10.8	7.4/7.9	

Pubis

LD0002

Element	Total length	Length of pubic plate	Length of pubic apron	Minimum transverse width of pubic apron	Depth of distal pubic apron expansion	Transvers width of distal pubic apron
LFGT-D0002						
Left				12*	8.8*	13.8*
Right					9.1	13.7
LFGT-D0003						
Left	41.2*	17.2	24.9	10.5*	6.7*	14.2*
Right					6.8	13*

Ischium

LFGT-D0002

Element	Height of distal end
Left	11
Right	11

Femur

Element	Total length	Mediolateral width of proximal end	Maximum proximodistal depth of femoral head	Proximal end to distal margin of 4th trochanter	Proximodistal length of 4th trochanter	Midshaft mediolateral width	Midshaft anteroposterior width	Transverse width of the distal end	Anteroposterior length of the distal end
LFGT-D0001									
Left					7.2	9.5	8.8	16.8	7.5
Right					6.3	8.4	6.9	17.2	6.8
LFGT-D0002									
Left	56	16.2	7.1	29	6.3*	9.4	8.8		
Right	61.4*	18.4	7.4	31.1	10	10.1	7.8	21.2*	8.4
LFGT-D0003									
left		19.2	7.1		7.2	9.5	8.8	16.8	7.5

Tibila and fibula

LD0001

Element	Total length	Anteroposterior length of proximal end	Transverse width of proximal end	Minimum width of midshaft (mediolateral)	Minimum length of midshaft (anteroposterior)	Anteroposterior length of distal end	Transverse width of distal end
LFGT-D0001							
Left tibia			13.8	5.6	5.8	7.3	13.4
LFGT-D0002							
Left tibia		16.7*	16*	6.3	6.9	8.4*	16.4*
Left fibula						9.8	5.3
Right tibia		17.5	16.4			8.9	
Right fibula		14.8					
LFGT-D0003							
Left tibia		15.8*	14.6*			7.7	16
Right tibia	43.9*	15.5*		5.6	6.6	7.6*	
Left fibula		14.4		4	4.4		
Right fibula				4.1	4.2	9.6*	5.4*

Astragalus and calcanum

Element	Dorsoventral height	Mediolateral width	Anteroposterior length
LFGT-D0001			
right astragalus	5.1	13.5	7.3
LFGT-D0002			
left astragalus		14.2	8.2
Left calcanum		5.1	4.7

Metatarsals and pedal phalanges

LFGT-D0002

Element	Total length (medial/lateral)	Proximal width (mediolateral)	Proximal height (dorsoventrally)	Minimum width of midshaft	Distal width (mediolateral)	Distal height (dorsoventrally)
Left Mt I	12.5	6.8	3.7	4.8*	5.2	4.2
Left Mt II	17.1/18.3	6.8*	7.8	3.9	5.9	3.8
Left Mt III	20.9/21.1		6.7	3.5	5.6	3.9
Left Mt IV	20.9*	8.3	2.8	2.9	3	3.9
Left Mt V	10.6	9	2.4	2	2.5	2.6
Right Mt I	10.1/11.2	7.8	4.7	4.9	6.4	4
Right Mt II	14.9/15.5	6.3*		4.1	6.9*	4.4
Right Mt V	10.4	8.9	1.4	2.1	2.3	2.7
Left Pl I-1	4.5/5.6	4.5*	4.4*	2.7*	3.4	4
Left Pl I-2	10.1	3.5	5.0			
Left Pl II-1	6.3/5.2	5.3	4.3	3.5	4.4	3.3
Left Pl II-2	4.6/3.8	4.2	4.4	3.5	3.5	3.3
Left Pl II-3	8.9/6.8	3.6	4.1			
Left Pl III-1	6.1/4.4/6.7	5.7	4.2	3.7	4.7	3
Left Pl III-2	3.2/2.8	4.1	3.6	3.6	4.2	2.8
Left Pl III-3	3.5/2.3	3.6	3			
Left Pl III-4	7.2/6.8	3	3			
Left Pl IV-1	4/3.6/5	4.6	4.1	3.5	4.2	2.2
Left Pl IV-2	3.4*	4.2	2.4	3.0	3.4	2.1
Left Pl IV-3	2.7	3.0	2.1	2.2	2.5	2.0
Left Pl IV-4	2.4	2.5	2.0	2.0	2.1	1.9
Left Pl IV-5	5.9/5.4		2.4			

Left Pl V-1	2.3	1.9	2.6			
Right Pl I-1	4.8/4.1		4.2	3.1	3.7	3.1
Right Pl I-2		3.5	5.1*			
Right Pl II-1	5.1/4.8		4.9	3.6*	4.8	3.5
Right Pl II-2	3.3/3.3	4.7	4.4	4	3.8	3.1
Right Pl II-3		3.5	4.2			
Right Pl III-1	4.7/5.6/5.5	6.1	4.1	4.8	5.7	3.3
Right Pl III-2	4.3/3.3/4.3	4.8				
Right Pl III-3	3.8	3.1*	3.4*		4*	2.4
Right Pl III-4		3.4	3.2			
Right Pl IV-1	4.2/4.7	4.6	3.4	3.5	4.1	2.2
Right Pl IV-2	2.8/3	3.9	2.6	3	3.5	1.9
Right Pl IV-3	2.2/2.3	3.2	2.3	2.5	2.8	1.7
Right Pl IV-4	1.8/2	2.8	2	2.1*	2.2	1.6
Right Pl IV-5		2.3	2.3			
Right Pl V-1	2.3	1.8	2.1			

1 McPhee, B. W., Bonnan, M. F., Yates, A. M., Neveling, J. & Choiniere, J. N. A new basal sauropod from the pre-Toarcian Jurassic of South Africa: evidence of niche-partitioning at the sauropodomorph–sauropod boundary? *Scientific Reports* **5**, 13224 (2015).
